# Books and Authors

**Published:** 1908-02

**Authors:** 


### BOOKS AND AUTHORS.

**Diseases of the Stomach.** By **Dr. I. Boas,** Berlin, Germany. Authorised English-American Edition by **Albert Bernheim,** M.D. Illustrated with five full-page plates and sixty-five engravings in the text. 730 royal octavo pages. Philadelphia: F. A. Davis Company. (Cloth $5.50; half morocco, $7.00.)

This author has been recognised for some years as an expert in gastro-enteric diseases; indeed, scarcely a treatise or monograph relating to this branch of medicine fails to quote Boas upon some technical or doubtful point. He is an acknowledged authority upon diseases of the stomach and speaks from the chair upon most questions of doubtful or disputed issue. An essential reason for conceding this high position to Boas, is because his writings are clear and concise statements of his own opinion, resulting from his own experience and frequently developed from his own research work. He never hesitates, but speaks like the master which he admittedly is.

American physicians will welcome this translation which has been made so admirably by Dr. Bernheim. It places within easy reach of the general practitioner who may not read German, a classic treatise that he can ill afford to do without.

The importance of a knowledge of diseases of the stomach has increased in direct ratio to the changes in our civilisation, and these have been so rapid in the last thirty years as to make us draw heavily on our energising apparatus. As a consequence, stomachal digestion has been taxed beyond its capacity in many instances. Breakdowns often begin at the stomach, though manifesting their effects elsewhere. The physician must be prepared to read the signs aright in the beginning; he must learn to diagnosticate correctly. This treatise will be of vast assistance to him in this respect, for Boas is first of all a diagnostican, and his directions relating to the examination of the patient, including the chemical examinations, the examination of the gastric contents, the urine, and the blood, comprise one-third of the book. Nothing could be more complete in its detail than this section and it should be studied with care by those who treat these diseases.

Under general therapeutics Boas first takes up the question of diet which he deals with in detail. No doubt many persons are finical regarding their food; on the other hand, it is important to know which articles of diet disagree with certain stomachs, hence a careful study of the effect of each article ingested on the offended stomach must be made. Valuable directions are given for such investigations. Lavage is another method thoroughly handled. No doubt this valuable aid is often abused, but this author teaches how to use it conservatively.

Several chapters are devoted to the consideration of special diagnostics and therapeutics which are full of interest, round ulcer and cancer being specially well handled. The book contains sixty-five illustrations in the text and five full-page plates. The engravings might be increased in number with advantage. An index of subjects and another of authors closes this most instructive as well as interesting treatise.

---

**Textbook of Obstetrics.** By **J. Whitridge Williams,** Professor of Obstetrics in Johns Hopkins University. Second Edition. Octavo, pp. 978. Illustrated. New York and London: D. Appleton and Company. 1908. (Cloth, $6.00.)

The first edition of this work was so well received by the profession that 17,500 copies were sold. We recall the delight of the JOURNAL when that book came to it, and we are even more pleased to receive this new copy, because of its freshness, and its enlarged condition, several new chapters having been added. These relate to the metabolism of normal pregnancy, vaginal Cesarean section, pubiotomy, and contractions of the pelvic outlet. The chapters on the development of the ovum, and upon the toxemias of pregnancy have been rewritten, and the entire book has been revised. It, therefore, has been reset and printed from new plates making this practically a new work.

Williams presents a thoroughly practical treatise founded upon a large experience as an obstetric teacher and clinician. We wish every teacher and author, for example, would give as exact a description as he does of Credé's method of delivering the placenta. This scientific bit of mechanism, correctly done, has saved thousands of lives; but it is astonishing how carelessly it is taught and how awkwardly it is described by the larger proportion of candidates for state license. Just so, too, with Credé's method of preventing ophthalmia neonatorum, as has been pointed out by F. Park Lewis, of Buffalo. Even Williams does not complete his description of the latter method, for he fails to mention that Crede drops the silver solution in the eyes from a glass rod ⅛ of an inch in diameter,—a very important part of the technic. If this method is adopted it will be impossible to do harm to the eyes.

The puerperium is well handled by Williams; so, too, are the infections. Operative obstetrics is an important field well dealt with; and, indeed, it is difficult to mention except to praise any portion of the treatise. We could wish diphthongs had been eliminated, and that labor had been spelled without the "u". This, of course, may be called pedagogic, but in these days simplicity is important.

The book is profusely illustrated with the better kind of engravings and is, all things considered, one of the best works on obstetrics of modern times.

---

**The Diagnosis and Treatment of Diseases of Women,** by **Harry Sturgeon Crossen,** M.D., Clinical Professor of Gynecology in Washington University; Gynecologist to Washington University and Chief of the Gynecological Clinic. Octavo, pp. 813. With 700 illustrations. Saint Louis: C. V. Mosby Medical Book and Publishing Company. 1907.

The literature of gynecology, already somewhat amply supplied as to quantity, is greatly enriched in quality by the publication of this work. It should be noted at the outset that its field is limited to the diagnosis and treatment of diseases of women. It is not concerned, except indirectly, with questions of etiology, pathology, or of major operative technic,—so the author states in his preface. This gives a wider opportunity for the consideration of those topics that have the greater interest for the general practitioner who must needs deal, more or less, with gynecological patients. It is he for whom the book is intended in particular and it is he who should make haste to obtain it for he will find it an invaluable guide to the work he is called upon to do.

It is not to be inferred from this that such a practitioner is not to familiarise himself with conditions requiring major operations for their relief, for this is by no means the case, the teachings of this book being quite to the contrary. It instructs as to the symptomatology of such conditions and of the kind of surgical intervention required; it is, indeed, to all intents and purposes a gynecological treatise of the first order with special presentation of questions relating to diagnosis and treatment.

Crossen presents a most complete and painstaking account of methods of examination of the patient in his first chapter, which comprises 117 pages. It is a surprising example of thoroughness and deserves unstinted praise for its perfection. Singularly enough this chapter contains 117 illustrations,—an average of one picture for each page of text. In the chapter on gynecologic diagnosis, too, there is marked precision of method. This chapter comprises 185 pages and contains 331 illustrations. We regard these two chapters as the most important portion of the book. In the third chapter various forms of treatment are described, considerable space being given to the tampon and pessary. Several cuts illustrative of the introduction of the pessary are given, but the simplest and best method of introducing this instrument is with the patient in the knee-chest posture, it being thus devoid of pain and capable of perfect adjustment.

The next chapter considers diseases of the external genitals and vagina, and gives points in anatomy. The fifth chapter deals with lacerations and fistulæ, the sixth with diseases of the uterus, the seventh with displacements of the uterus, the eighth with non-malignant tumors of the uterus, the ninth with malignant disease of the uterus, the tenth with pelvic inflammation, the eleventh with other affections of the Fallopian tubes, pelvic peritoneum and connective tissue, the twelfth with tumors of the ovary and parovarium, the thirteenth with malformations, the fourteenth with disturbances of function, the fifteenth with invasion of the peritoneal cavity, the sixteenth with the after-treatment in operative cases, and the seventeenth with medico-legal points in gynecology.

An appendix follows containing many useful formulæ for both local and general use, and an index concludes this valuable book. Among the final chapters we desire to commend in particular the one dealing with the invasion of the peritoneal cavity. Many of the details given are not found in textbooks. Of the seven hundred illustrations which the book contains two hundred and twenty and more are original, showing the author's own methods. It is one of the more important books of the year 1907, and gives evidence of skilful and painstaking effort on the part of the author.

---

**A Textbook of Practical Diagnosis.** By **Hobart Amory Hare,** M.D., Professor of Therapeutics in the Jefferson Medical College of Philadelphia. Sixth edition. Octavo, 616 pages, with 203 engravings and 16 full-page plates. Lea Brothers & Co., Philadelphia and New York, 1907. (Cloth, $4.50; leather, $5.50, net prices.)

Diagnosis at the present day consists of a combination both of clinical examination and laboratory investigation. The physical examination including history and analysis of symptoms, is as important now as ever, notwithstanding the strides recently made by laboratory methods in the investigation of disease. Hare combines the two when necessary, but very properly places the clinical facts first. In the presence of the patient, whether in the consulting room or at the bedside, the physician should be able to elicit the most important information from the symptoms presented; the blood and urine may be appealed to when indicated as they always are by intelligent practitioners.

Hare states his position in a nutshell when, in his preface, he says "this book is written upon the only plan that can be followed in practice—namely, the up-building of a diagnosis by grouping the symptoms." This sixth edition has been considerably enlarged and is a companion to the author's textbook of practical therapeutics which appears in its twelfth edition simultaneously with this book. Also, it may be noted that Hare's treatise on the practice of medicine has passed recently to its second edition. These three books, together constitute a remarkable triad and give to a physician some of the best litterature extant relating to the diagnosis and treatment of disease.

---

**A Treatise on Fractures and Dislocations.** By **Lewis A. Stimson**, B.A., M.D., Professor of Surgery in Cornell University Medical College, New York. Fifth edition. Octavo, 847 pages, with 352 engravings and 52 plates. Lea Brothers & Co., Philadelphia and New York, 1907. (Cloth, $5.00, leather, $6.00, half morocco, $6.50, net prices.)

This standard treatise, by an author recognised everywhere for his learning and surgical skill, will be welcomed by all surgeons in its fifth edition. Stimson is well fitted to deal with these topics—fractures and dislocations,—being a methodical man himself; and none other can effectively treat these injuries. A mechanical eye and a deft hand is here needed, if ever, and this author has both, which he is capable, too, of using to the best advantage.

Moreover, these are emergency traumatisms which make sudden demand on the resources of the surgeon and Stimson has impressed this fact throughout his book. Most persons, especially those of the uneducated class, expect perfect results in case of fractured bones, and usually blame the surgeon if these conditions are not obtained. It is better to inform such persons at once that no surgeon can restore the parts to the perfection that nature established. If, however, the teachings of this book are followed, most bone and joint injuries will be sufficiently restored to their original state for all practical purposes. This will be the case with the fractures of long bones in particular.

Whatever additional knowledge the *x*-rays have afforded in the study of fractures, especially in the diagnosis of old fractured joints, has been made use of in this treatise. The illustrations are numerous and excellent in quality. We may add, in conclusion, that the entire treatise represents the modern treatment of the injuries with which it deals.

---

**A Textbook of Clinical Anatomy.** By **Daniel N. Eisendrath,** A.B., M.D., Adjunct Professor of Surgery in the Medical Department of the University of Illinois. Octavo, pp. 535. Illustrated. Second edition. Philadelphia and London: W. B. Saunders Company. (Price, $5.00.)

Though anatomy is an old subject, yet it is presented in this work on new lines of study. Four years ago when it was first issued no similar anatomical studies had been presented in a single treatise. It teaches surface and topographic anatomy in a manner not found in any other work, and it applies anatomic landmarks to the clinical exposition of medical and surgical disease. It is a book nearly as necessary for the physician as for the surgeon. It is as important, also, for the advanced student to become familiar with its teachings as for the practitioner to know the methods of diagnosis.

One of the important functions of this work is to refresh the memory of the general practitioner in regard to anatomy. It is better for this purpose than a general descriptive anatomy, being less loaded with details and clearer and more concise in describing regional anatomy. Again, it encourages the study of anatomy on the living subject,—the anatomy of life,—which is clinical anatomy in fact as well as in name.

For these, as well as for many other reasons not necessary to name, we regard this one of the most important works on anatomy yet issued,—an anatomy for the advanced student, for the general practitioner, for the specialist, and for the surgeon.

---

**Practical Fever Nursing.** By **Edward C. Register**, M.D., Professor of the Practice of Medicine in the North Carolina Medical College. Octavo, pp. 352. Illustrated. Philadelphia and London: 1907. W. B. Saunders Co. (Price, $2.50.)

This book appears to be half treatise on practice and half on nursing. It is too small for one and not quite large enough for both. Nevertheless, it contains much that is useful and may receive the attention of physicians who, themselves, may be uninstructed as to the duties a nurse should perform for a fever patient. The advanced nurse, or the nurse who has not had adequate hospital training may gain valuable information by a study of the book. The literature relating to nursing is becoming somewhat voluminuous, and we doubt the expediency of further adding to the burden of the already overtaxed nurse, by asking her to obtain books that are not absolutely necessary.

---

**American Edition of Nothnagel's Practice.** Diseases of the Intestines and Peritoneum. Second edition. Edited by **Alfred Stengel,** M.D., Philadelphia. Philadelphia and London: W. B. Saunders Co. (Price, cloth, $5.00; sheep or half morocco, $6.50.)

It is an agreeable reflection on the desire of the American medical profession for good literature, that a new edition of Professor Nothnagel's practice should be required so soon, it being less than four years since the first edition was published. It is gratifying, too, to observe that this second edition is put forth under the same auspices as the first,—the same editors and publishers being responsible for this issue.

A remarkable fact relating to this work is that it should require more than one thousand pages of standard octavo text to deal with diseases of the intestines and peritoneum,—more pages of text, indeed, than an entire treatise on the theory and practice of medicine required fifty years ago. This indicates the progress making in perfecting the diagnosis and treatment of diseases in general as well as those in particular with which this work deals.

The English editor, Dr. Rolleston, has made many additions and some alterations of the arrangement since the edition of 1904 was published. The text, however, of the author remains the same, the additions being included in brackets. Those who failed to obtain the first edition of the American translation will have an opportunity now to possess themselves of this great work with the latest annotations and references, making it strictly a modern treatise in every respect.

---

**Diseases of the Rectum.** Their Consequences and Nonsurgical Treatment. By **W. C. Brinkerhoff,** M.D. 12 mo., pp. 207. Chicago: Orban Publishing Co. (Price, $2.00.)

Whatever this book may lack in scientific research it makes up in an endeavor to become controversial in its nature. The author advocates the injection treatment for hemorrhoids; he gives useless conversation with patients; talks about the Brinkerhoff injection system of treatment for hemorrhoids as though it were something novel, and ends his book with a tirade against state examinations. If this book has any scientific value or professional interest we should be glad to have them pointed out.

---

**Manual of the Diseases of the Eye.** By **Charles H. May,** M.D., Instructor in Ophthalmology, College of Physicians and Surgeons, New York. Fifth edition. 12 mo, pp. 391. Illustrated. New York: William Wood & Co. (Price, $2.00.)

It is a little over two years since the fourth edition of this excellent manual was issued. In that time some advances have been made in relation to diseases of the eye, which May has incorporated in this revision, every page of which has been carefully examined with this end in view. The illustrations have been changed somewhat, but the book remains the same in size and remains now, as it always has been, one of the better of the small eye manuals.

---

**The Operating Room and the Patient.** By **Russell S. Fowler,** M.D., Professor of Surgery, Brooklyn Post-Graduate Medical School. Second edition. Octavo, 284 pages. Illustrated. Philadelphia and London: W. B. Saunders Company, 1907. (Cloth, $2.00 net.)

The appearance of this book two years ago was gratifying to surgeons and especially, perhaps, to those interested in the practice of surgery, though themselves not distinctly specialists in surgery. To have a manual at hand giving full instructions for the preparation of patient and operating room is most opportune. Important interlocutory questions also are dealt with in this volume, such as the administration of the anesthetic, the after-treatment, lists of various instruments and appliances used in different operations, and so forth, which increases its value. In this edition are chapters on general considerations of after-treatment, wound complications and the like, thus adding to its usefulness as a guide to the young surgeon and operators assistant. Nurses, too, will find the book of value in the preparation of patients for the surgeon's work. It is a book that should find its way easily to the table of everyone interested in surgical work.

